# Dr. Charles McBurney

**Published:** 1913-11-29

**Authors:** 


### Obituary.

Dr. Charles McBurney, the American doctor, known to science as the inventor of McBurney's point—that is, the discoverer of the exact position of pain caused by pressure in the preliminary development of appendicitis, has died at the age of sixty-eight. His high position as a medical authority in the States is testified by the positions which he held: Professor of Clinical Surgery in the College of Physicians and Surgeons, New York, and Visiting Surgeon to the Roosevelt Hospital.

